# Recurrent squamous cell carcinoma arising in ovary mature cystic teratoma: A case report

**DOI:** 10.1097/MD.0000000000034734

**Published:** 2023-08-11

**Authors:** Xuechao Ji, Peiling Zhai, Hanchao Yang, Hui Wang, Xinbo Wang

**Affiliations:** a Department of Obstetrics and Gynecology, Affiliated Hospital of Weifang Medical University, Weifang, Shandong, China; b Department of Gynecologic Oncology, Beijing Obstetrics and Gynecology Hospital, Capital Medical University, Beijing Maternal and Child Health Care Hospital, Beijing, China; c Department of Pathology, Affiliated Hospital of Weifang Medical University, Weifang, Shandong, China.

**Keywords:** chemotherapy, cytoreductive surgery, mature cystic teratoma, recurrence, squamous cell carcinoma

## Abstract

Malignant transformation of mature cystic teratoma is very rare, of which squamous cell carcinoma (SCC) is the most common type. Prognosis of SCC arising in mature cystic teratoma of the ovary is very poor. Our experience may provide new ideas for the treatment of this disease.

**Patient concerns:**

The patient was a 56-year-old woman and was admitted for a lower abdominal pain. She underwent a laparoscopic surgery with 4 cycles of chemotherapy and had achieved a complete response; 10 months after the completion of initial treatment, her cancer relapsed. She underwent a cytoreductive surgery with concurrent chemoradiotherapy and has achieved a complete response again.

**Diagnoses:**

This patient was initially diagnosed with ovarian cancer (stage IIIB) arising from malignant transformation of mature teratoma; 10 months after the completion of initial treatment, she was diagnosed with recurrent ovarian cancer.

**Interventions:**

This patient was initially treated with laparoscopic bilateral salpingo-oophorectomy. After histopathological confirmation that she had ovarian cancer, she underwent laparoscopic total hysterectomy and omentectomy with 4 cycles of chemotherapy. After her ovarian cancer recurred, she underwent open cytoreductive surgery and concurrent chemoradiotherapy.

**Outcomes:**

The patient achieved complete response after both initial and relapsed treatment.

**Lessons:**

Optimal cytoreduction and concurrent chemoradiotherapy may be an option to improve the prognosis of patients with recurrent SCC arising in ovary mature cystic teratoma.

## 1. Introduction

Ovarian teratoma is a common tumor of the female reproductive system, which mostly occurs in women of childbearing age. Most ovarian mature cystic terotomas (MCT) are benign. Malignant transformation of mature cystic teratoma (MT-MCT) is very rare, with a malignant transformation rate of 0.17% to 2%.^[1,2]^ The 5-year survival rate of advanced MT-MCT is 0.^[3]^ In this article, we report a case of recurrent squamous cell carcinoma (SCC) arising in ovary MCT.

## 2. Patient information

**Figure 1.**
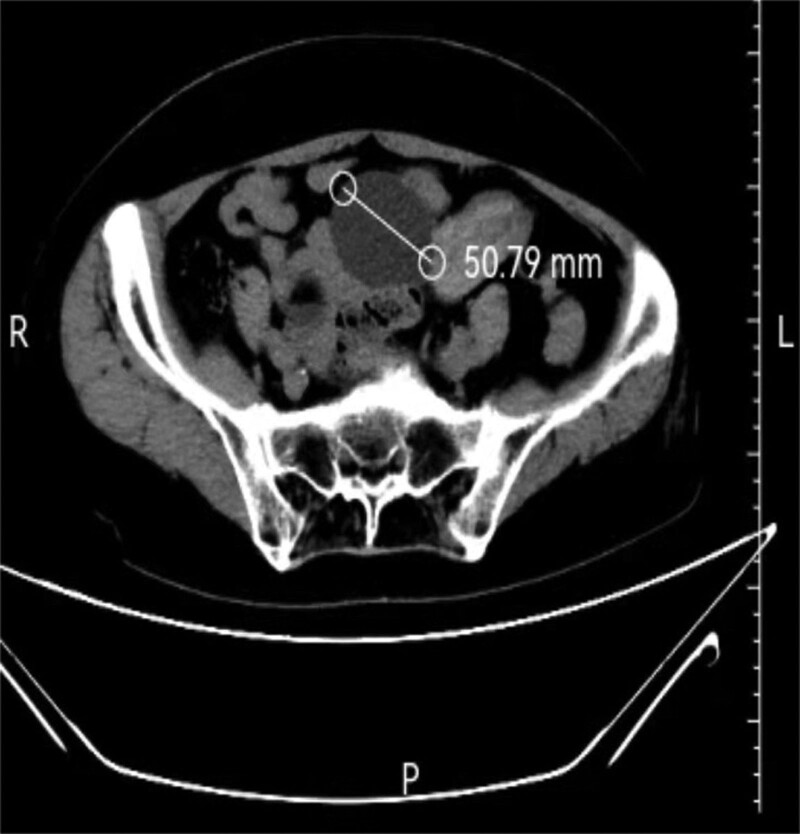
Abdominal computer tomography scan: a triple tissular mass with greasy and osseous constituent (=85 mm).

**Figure 2.**
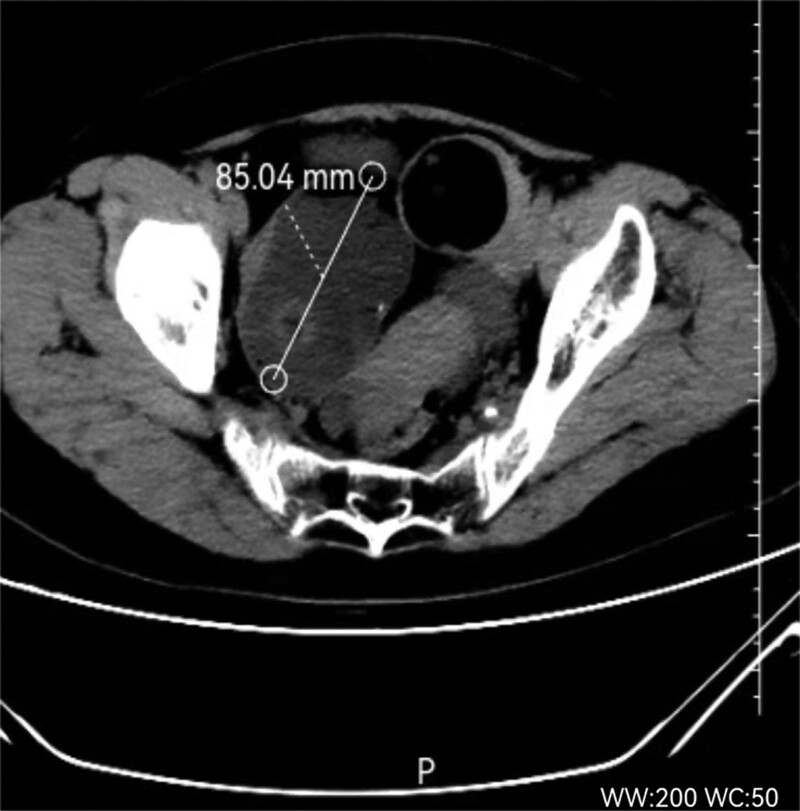
Abdominal computer tomography scan: a triple tissular mass with greasy and osseous constituent (=50 mm).

**Figure 3.**
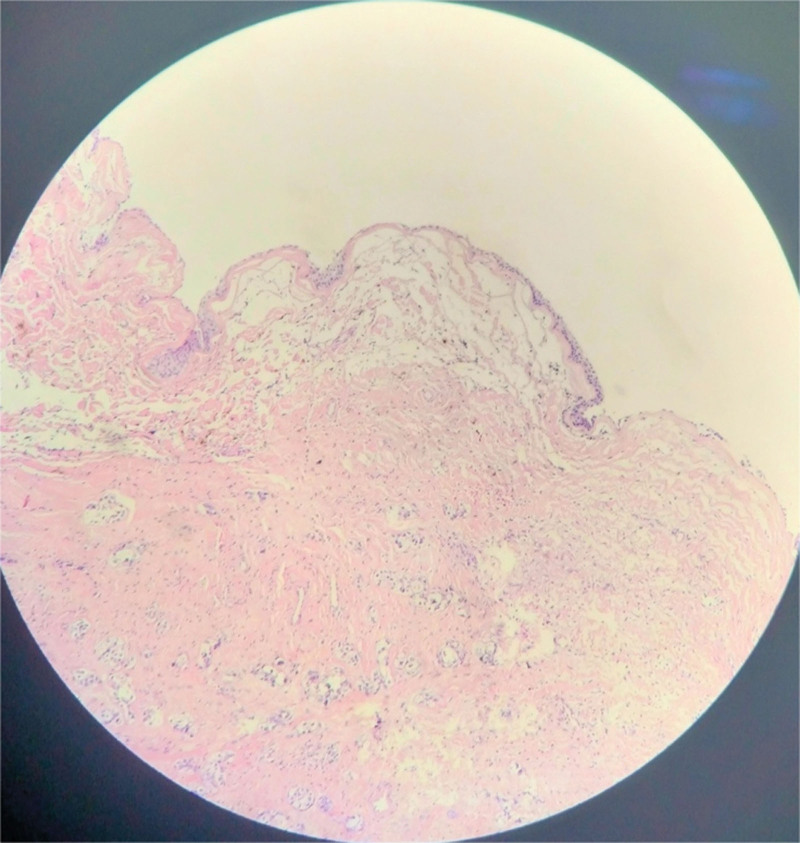
Squamous cell carcinoma in situ (G*100).

A 56-year-old, postmenopausal woman was admitted to our gynecologic emergency for lower abdominal pain, with a 10-year history of ovarian teratoma, which was suspected to be a torsion of ovarian tumor at first. Abdominal computed tomography scan revealed bilateral ovarian tumors with fat and soft tissue components. The diameters of the left ovarian tumor were 50 and 60 cm (Fig. 1), and the diameter of the right ovarian tumor was 85 mm (Fig. 2). The serum tumor marker levels were as follows: carbohydrate antigen (CA) 125: 21.11 U/mL, CA 19-9: 92.79 U/mL, carcinoembryonic antigen: 5.57 ng/mL, alpha fetal protein: 1.92 ng/mL, estradiol <5 pg/mL, human epididymis protein 4: 36.98 pmol/L. This patient underwent bilateral salpingo-oophorectomy. The frozen pathology analysis during the operation showed that the right ovarian teratoma, with a certain amount of atypical epithelial cells, was found in the fibrous tissue of the cyst wall. The patient family members strongly requested that the uterus be retained. Postoperative histopathological analysis revealed that the right ovarian MCT, with locally malignant transformation into SCC (Fig. 3). Then she underwent a total hysterectomy and omentectomy. The histopathological analysis revealed that squamous cell cancer tissue was found in omentum, with a diameter of 10mm. She was diagnosed with ovarian cancer (International Federation of Gynecology and Obstetrics stage IIIB). After second surgery, she underwent 4 cycles of chemotherapy, which contained bleomycin, etoposide and cisplatin. According to the response evaluation criteria in solid tumors, she had achieved a complete response.

**Figure 4.**
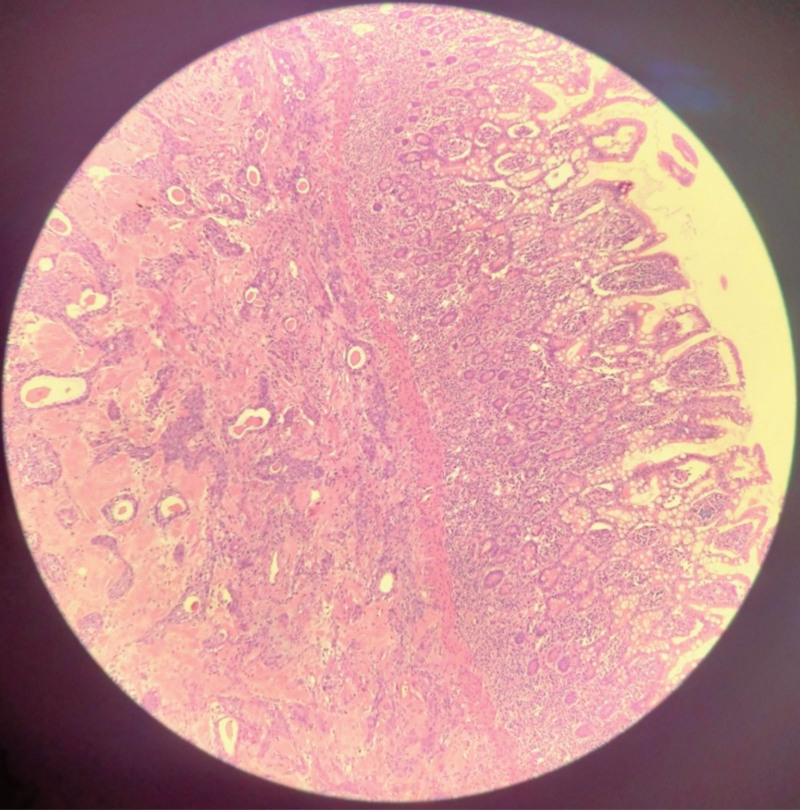
Squamous cell carcinoma invading small intestine (G*100).

10 months after the completion of initial treatment, the patient found a periumbilical tumor. The abdominal computed tomography scan revealed multiple soft tissue nodules in the lower abdominal wall. Serum tumor markers were as follows: squamous cell carcinoma antigen (SCCA): 6.67 ng/mL, CA 19-9: 33.54 U/mL, CA 125: 6.05 U/mL, alpha fetal protein: 2.7 ng/mL. This suggested that the patient had tumor recurrence and metastasis. The patient underwent a tumor cytoreductive surgery, including pelvic and abdominal tumor resection, appendectomy, partial intestinal resection and anastomosis, periumbilical tumor and navel resection, as well as right ureteral D-J tube insertion through ureteroscope. Postoperative histopathological analysis showed poorly differentiated squamous cell carcinoma was found in the umbilicus, vaginal stump, bladder muscle layer, small intestine, mesocolon, colon, appendix and anterior rectum (Fig. 4). Then she underwent concurrent radiochemotherapy, the chemotherapy regimen was paclitaxel and nedaplatin, and the radiotherapy regimen was abdominal external radiotherapy. The patient achieved a complete response again.

Timeline

**Table:** 

The event	Timeline
Symptom of lower abdominal pain	February 2021
A laparoscopic bilateral salpingo-oophorectomy was performed	February 2021
A total hysterectomy and omentectomy were performed	March 2021
Chemotherapy	March 2021–May 2021
Recurrence and metastasis	March 2022
A cytoreductive surgery was performed	March 2022
Concurrent radiochemotherapy	March 2022–June 2022
Follow-up showed no recurrence or metastasis	April 2023

## 3. Discussion

Ovarian MCT is a common benign tumor, accounting for 10% to 20% of all ovarian tumors. 10% to 17% of the reported cases were bilateral ovarian tumors.^[4,5]^The peak onset age of MCT is between 30 and 40 years old.^[6]^ The incidence rate of ovarian MCT is 1.2 to 14.2 cases per 100,000 cases per year, and the proportion of cases with malignant transformation is 0.17% to 2%.^[7–9]^ The prognosis of patients with advanced MT-MCT is very poor. The 5-year survival rate is 94.7% and 80.0% for patients with stage I and II, respectively, but 0 for patients with stage III and IV.^[3]^ Mature teratomas could transform into SCC, adenocarcinoma, adenosquamous carcinoma, carcinoid, etc. The most common type of malignant transformation is SCC, accounting for about 80% of MT-MCT.^[1,6]^ However, the cause of MT-MCT is still unclear, and some scholars believe that high-risk HPV infection may be one of the causes.^[10]^

The onset age of MT-MCT patients is older than that of benign MCT patients, often over 45-year-old. Patients were often with a history of teratoma. The diameter of MT-MCT usually exceed 10 cm in diameter. The level of SCCA may be elevated. However, MT-MCT has no specific clinical manifestations, most patients have no symptoms, only a few patients show abdominal distension or abdominal mass. There is no obvious change in imaging examination of early-stage patients, and there are no specific serum tumor markers. Some scholars have suggested that the probability of malignant transformation of MCT into SCC increases when the patient is in menopause or older than 45 years,^[11]^ the tumor diameter exceeds 10 cm,^[11,12]^ ultrasound indicates that tumor blood supply is high-frequency and low impedance signal,^[13]^ with serum SCCA exceeding 2.5 ng/mL.^[14]^ Others believe that serum macrophage colony-stimulating factor, tumor antigen 4, tissue polypeptide antigen and other tumor markers can also be used to differentiate benign and malignant MCT.^[15–17]^

In view of the low incidence of MT-MCT, there is no standard treatment for MT-MCT. Most scholars believe that MT-MCT should refer to the treatment of ovarian cancer, and surgery is the first choice.^[3,12,18,19]^ Relevant auxiliary examinations should be comprehensively implemented before surgery. Early-stage patients should undergo comprehensive staging surgery, and advanced stage patients should achieve optimal cytoreduction.^[20–22]^ After surgery, according to the stage of International Federation of Gynecology and Obstetrics, adjuvant therapy of platinum-based chemotherapy programs for epithelial ovarian cancer is commonly used at present, such as paclitaxel and carboplatin or paclitaxel and cisplatin.^[20,21,23,24]^ The effect and feasibility of radiotherapy remains controversial.^[25]^ Some scholars believe that if early-stage patients have completed comprehensive staging surgery, they can choose to be observed, and advanced stage patients should receive adjuvant treatment after cytoreduction surgery.^[19–21]^ The therapeutic effect of postoperative chemotherapy may be better than that of radiotherapy.^[21]^

MT-MCT originates from germ cells. Chemotherapy regimen for ovarian germ cell tumors, such as bleomycin, etoposide and cisplatin, may be useful. For patients with relapse or drug resistance, the treatment scheme of SCC in other organs can be considered, such as chemotherapy with 5-fluorouracil and folic acid, combined with radiotherapy or concurrent chemoradiotherapy.^[26]^ If traditional treatment methods are invalid, gene, immune status and molecular targeting detection should be considered. Some scholars have confirmed that MT-MCT has mutations in TP53, P16, MLH1, and other gene sites, and several patients have already benefited from immunotherapy and anti-angiogenic therapy.^[27–30]^ However, there is no large-scale clinical trial of MT-MCT treatment at present, and the therapeutic effect of the above methods remains unclear.

The prognosis of early and advanced stage MT-MCT has enormous difference. Some researchers believe that the high-risk factors including age, tumor stage, histological differentiation, tumor marker level could predict the prognosis.^[3,6,12,20,23,24]^ To improve the prognosis of the high-risk patients, more progressive treatments should be considered.

According to this case, complete surgical resection is essential for the management of MT-MCT. For the choice of chemotherapy regimen, the use of SCC or germ cell tumor chemotherapy can be considered. New treatment options such as radiotherapy and targeted therapy should also be considered.

## Author contributions

**Conceptualization:** Peiling Zhai.

**Data curation:** Peiling Zhai, Hanchao Yang.

**Funding acquisition:** Xinbo Wang.

**Resources:** Hanchao Yang.

**Validation:** Peiling Zhai, Hui Wang.

**Writing – original draft:** Xuechao Ji, Hui Wang.

**Writing – review & editing:** Hanchao Yang, Xinbo Wang.
